# SMG9 Serves as an Oncogene to Promote the Tumor Progression *via* EMT and Wnt/β-Catenin Signaling Pathway in Hepatocellular Carcinoma

**DOI:** 10.3389/fphar.2021.701454

**Published:** 2021-08-12

**Authors:** Xing Jin, Jie Yin, Hongling Zhu, Weikang Li, Kewei Yu, Miao Liu, Xiujuan Zhang, Miaolian Lu, Zemin Wan, Xianzhang Huang

**Affiliations:** ^1^ Second Clinical College, Guangzhou University of Chinese Medicine, Guangzhou, China; ^2^ Department of Laboratory Medicine, The Second Affiliated Hospital of Guangzhou University of Chinese Medicine, Guangzhou, China; ^3^ Department of Nuclear Medicine, The Affiliated Hospital of Yangzhou University, Yangzhou, China; ^4^ Department of Gynecology, Shanghai Armed Police Corps Hospital, Shanghai, China; ^5^ Department of Liver Disease, The Second Affiliated Hospital of Guangzhou University of Chinese Medicine, Guangzhou, China

**Keywords:** SMG9, hepatocellular carcinoma, EMT, prognostic biomarker, therapeutic target

## Abstract

**Background/Aims:** SMG9 participates in the nonsense-mediated mRNA decay process that degrades mRNA harboring nonsense mutations introduced either at the level of transcription or RNA processing. However, little is known about the role of SMG9 in hepatocellular carcinoma (HCC). The objective of this research was to clarify the effects of SMG9 expression on HCC progression.

**Methods:** Microarray data were acquired from NCBI Gene Expression Omnibus (GEO) and The Cancer Genome Atlas (TCGA) database to bioinformatically analyze the differential expression of SMG9 between HCC patients and normal controls. SMG9 mRNA level was measured in sixteen sets of fresh tumor tissues and adjacent non-cancerous liver tissues (ANLTs) *via* reverse transcription-quantitative polymerase chain reaction (RT-qPCR). SMG9 protein expression was analyzed in ninety-five sets of paired formalin-fixed and paraffin-embedded tissue specimens by immunohistochemistry (IHC). In addition, clinicopathological features of SMG9 in HCC were checked. For *in vitro* studies, small interfering RNA (siRNA) was used to silence SMG9 expression for exploring biological functions *and* underlying *mechanisms* of SMG9 in SMMC-7721 and HepG2.

**Results:** We found that SMG9 was upregulated in HCC tissues and SMG9 levels were closely related to TNM stage, tumor number and tumor size. Cox regression and Kaplan–Meier proportional hazards analyses showed that high expression of SMG9 was associated with poor patient survival. Furthermore, proliferation, apoptosis resistance, migration and invasion of both SMMC-7721 and HepG2 cells were suppressed by SMG9 inhibition. In addition, EMT and the Wnt/β-catenin signaling pathway were involved in SMG9-mediated HCC progression.

**Conclusions:** SMG9 may serve as a potential novel prognostic biomarker and therapeutic target in HCC patients.

## Introduction

Hepatocellular carcinoma (HCC), as the principal histological type of liver cancer, comprises the great majority of liver cancer diagnoses and deaths (Arnold et al., 2020). The risk factors underlying HCC are highly variable, inducing genomic alterations, epigenetic changes and gene mutation, finally leading to HCC progression (Ding et al., 2019). To date, great efforts have been made in understanding the pathogenesis of HCC, and several preventive and therapeutic treatments have been applied in the management of HCC (Llovet et al., 2021). Unfortunately, the overall prognosis of HCC still remains dismal (Bray et al., 2018; McGlynn et al., 2021). Thus, discovering new prognostic biomarker and therapeutic target are still urgently needed to monitor the progression of HCC and improve the clinic outcomes of HCC.

The poor outcome of HCC is mainly due to its complexities, reoccurrence after surgical resection, metastasis and heterogeneity (Forner et al., 2018; Kabashima et al., 2021; Llovet et al., 2021; McGlynn et al., 2021). Recent studies have shown that communications between tumor cells and tumor microenvironment (TME) play key roles in promoting tumor progression (Sukowati et al., 2015; Lu et al., 2019). Among all the stromal cells in TME, cancer-associated fibroblasts (CAFs) are the major types to support and regulate tumor cell proliferation, apoptosis, migration, invasion, angiogenesis, immune escape, and drug resistance (Hernandez-Gea et al., 2013; Yin et al., 2018). CAFs are composed of a heterogeneous group of activated fibroblasts with different cellular origins (Yin et al., 2019). Tumor cells undergo the epithelial-mesenchymal transformation (EMT) is one of the well-established sources of CAFs in the TME (Giannelli et al., 2016). Therefore, targeting CAFs by reducing the EMT is an ideal strategy in suppressing tumor progression. However, there is no satisfied and efficient biomarker that could be used as the therapeutic target for inhibiting EMT in HCC progression.

Early HCC-related studies mainly focused on molecules derived from either transcriptomics or proteomics due to their central roles in the regulation of biological processes (Pei et al., 2009). In clinics, proteomic markers, such as alpha-fetoprotein (AFP) and des-gamma-carboxy prothrombin (DCP), are widely used diagnostic markers for HCC (Kanwal and Singal, 2019). However, their sensitivity and specificity remain limited. Recently, increasing evidences indicate that post-transcriptional modifications and remodeling also play key roles in regulating tumor progression, as cancers often exhibit abnormal transcription and mRNA processing (Lombardi et al., 2020). Nonsense-mediated decay (NMD) is a post-transcriptional surveillance mechanism that recognizes and degrades mRNAs containing premature translation termination codons (PTCs) to ensure the fidelity and accuracy of process from transcription of genetic information to protein synthesis (Yamashita et al., 2009). To date, the core factors involved in the NMD have been identified, and SMG9 is one of them (Fernandez et al., 2011). SMG9 is a 520-amino acid protein that contains a nucleotide triphosphatase domain in the center (Fernandez et al., 2011). SMG9 supports and stabilizes the SMG1 complex (SMG1C) formation by binding to SMG1 and SMG8, and involves in PTCs recognition, a key step for degradation of a PTC-containing mRNA (Deniaud et al., 2015). It has been well documented that cells cannot recognize transcripts containing PTCs with loss-of-function mutations of SMG9(Shaheen et al., 2016), suggesting the pivotal role of SMG9 in post-transcriptional regulation and surveillance. In addition, SMG9 deficiency has been reported to exhibit extensive transcriptional disorders and a number of malformations in human (Lecoquierre et al., 2019; Alzahrani et al., 2020). However, whether and how SMG9 involves in the initiation and progression of HCC remains unknown. Given the importance that SMG9 and NMD play in post-transcriptional regulation and surveillance, it is a valuable work to investigate the biological function of SMG9 in HCC.

In this study, we have demonstrated that SMG9 significantly increased in the tissues of HCC compared with the adjacent non-cancerous liver tissues (ANLTs), and high expression of SMG9 was associated with poor prognosis in the HCC patients. *Functionally*, SMG9 significantly promoted cell proliferation, cell cycle progression, apoptosis resistance, invasion and migration of HCC cell line. Mechanically, *these processes* might be driven by EMT and the Wnt/β-catenin signaling pathway. To sum up, our findings suggest that SMG9 is both a new promising prognostic biomarker and specific therapeutic target for HCC.

## Materials and Methods

### Specimen Collection

The liver tissues for this study were obtained from HCC patients who had undergone hepatectomy at The Affiliated Hospital of Yangzhou University (Yangzhou, China) from July 2013 to December 2014. Patient clinical characteristics are shown in Table 1. None of these patients were treated with chemotherapy or preoperative radiotherapy. All the patients were followed up until December 2020. All tissue specimens were made anonymously in accordance with legal and ethical standards. Fresh tumor tissue specimens were frozen in liquid nitrogen immediately after resection and kept at −80°C. In our study, overall survival (OS) is defined as the time from surgical operation to death or final contact for any reason, and disease free survival (DFS) is regarded to be the time from surgical operation to the first recurrence/metastasis of tumor or death due to any cause (Hou et al., 2019). Patients alive without tumor metastasis and recurrence were censored at the time of last follow-up.

**TABLE 1:** Correlations between SMG9 expression and clinical characteristics of HCC patients.

Variables	SMG9 expression		*p* value
	Low (N = 41)	High (N = 54)	
Sex			
Male	28	30	0.207
Female	13	24	
Age, years			
＜60	20	28	0.767
≥60	21	26	
AFP, ng/L			
＜400	22	28	0.861
≥400	19	26	
Tumor number			
Single	32	29	**0.014**
Multiple (≥2)	9	25	
Tumor Size, cm			
≥5	14	32	**0.015**
＜5	27	22	
TNM Stage			
I&II	27	23	**0.025**
III&IV	14	31	

Bold values indicate statistical significance. If the *p* value was <0.05, the data were considered to be statistically significant difference.

### High-Throughput Sequencing Data Analysis

We acquired HCC expression data from the Gene Expression Omnibus (GEO; http://www.ncbi.nlm.nih.gov/geo) and The Cancer Genome Atlas (TCGA; https://portal.gdc.cancer.gov/) database. The datasets GSE14520, GSE6764, GSE45436 and GSE51401 were downloaded from the GEO database (Wurmbach et al., 2007; Roessler et al., 2010; Wang et al., 2013). All data were transformed via log2 and analyzed using GraphPad Prism 7 and EdgeR software (packages for quasi-likelihood testing, generalized linear model, exact testing, empirical Bayes estimation, and negative binomial distribution). Overexpression of SMG9 is defined as a higher level of SMG9 expression in tumor tissues than that in normal tissues and the difference is statistically significant at *p* value <0.05. Genes were considered as differential expression genes at a *p* value <0.05 together with a Log fold change (Log FC)≤ −1.0 or ≥1.0.

### Immunohistochemistry

Paired formalin-fixed, paraffin-embedded tissue specimens from ninety-five cases of HCC were used for SMG9 immunohistochemistry (IHC) studies. HCC sections were deparaffinized with xylene and rehydrated in graded concentrations of ethanol. For antigen retrieval, sections were heated in a microwave oven for 15 min in 0.01 M citric saline (PH = 6.0). After incubating with 3% aqueous solution of hydrogen peroxide (H_2_O_2_) for 25 min in the dark, sections were further blocked in 5% goat serum (Beyotime, Shanghai, China) for 30 min at room temperature. Then sections were incubated with primary antibody against SMG9 (1:100; Abcam, Cambridge, United Kingdom) overnight at 4°C. Next day, after washing with phosphate buffered saline (PBS), the HCC sections were incubated with secondary antibody (Gene Tech, China) at room temperature for 30 min. The expression level of SMG9 in the tissues was visualized by diaminobenzidine (DAB) (Gene Tech, China) staining and re-dyed with hematoxylin for 5 min. Finally, the sections were sealed with neutral gum and photographed under a light microscope (Leica, Germany). Specimens were visually scored based on staining intensity (0, no staining; 1, slightly more yellow than the context; 2, yellow-brown; 3, brown) and percentage of positive cells (0, 0–5%; 1, 6–25%; 2, 26–50%; 3, 51–75%; 4, >75%).

The IHC score was calculated as follows: total score = staining intensity score × percentage of positive cell score. Four classifications were used: 0, negative (−) staining; 1–4, weak positive (+) staining; 5–8, positive (++) staining; 9–12, strong positive (+++) staining.

### RNA Extraction and Reverse Transcription-Quantitative Polymerase Chain Reaction

TRIzol reagent (Invitrogen, NY, United States) was used to extract total RNA according to the manufacturer’s instructions. An SYBR Green PCR Master Mix (Takara, Kyoto, Japan) was used to perform reverse transcription-quantitative polymerase chain reaction (RT-qPCR). The primers for SMG9 were as follows: anti-sense, 5′-TGC​AGC​CAT​GAT​ATG​AGC​GTC-3′; sense, 5′-GGA​TGA​ACA​GGC​TCT​ATT​AGG​GC-3′ (Servicebio Technology, Wuhan, China). GAPDH was used as an internal control, and the primers for GAPDH were as follows: reverse primer, 5′-ACA​CCA​TGT​ATT​CCG​GGT​CAA​T-3′; forward primer, 5′-TGT​GGG​CAT​CAA​TGG​ATT​TGG-3′ (Servicebio Technology, Wuhan, China). The relative mRNA expression levels of SMG9 were quantified by the 2^−ΔΔCt^ method. The RT-qPCR experiments were performed in triplicate and independently repeated three times.

### Cell Culture

SMCC-7721 and HepG2 human HCC cell lines were acquired from the American Type Culture Collection (ATCC, Manassas, VA) and grown in DMEM supplemented with 10% fetal bovine serum (FBS) containing 1% antibiotics (streptomycin and penicillin) at 37°C in a 5% (v/v) CO_2_ humidified environment.

### RNA Interference

SMMC-7721 and HepG2 cells were seeded in six-well plates at a density of 1 × 10^5^/ml before transfecting with a 50 nM concentration of small interfering RNAs (siRNAs) targeting SMG9 (Ribobio, Guangzhou, China) and negative control (NC) siRNA (Ribobio, Guangzhou, China). Transfection was performed using Lipofectamine™ 3,000 (Invitrogen, California, United States) referring to the instructions. The siRNA sequences are as follows:

• siRNA-1: GCA​CTT​CCG​TCA​TGC​AGA​A
• siRNA-2: GGA​GCC​AAG​TGA​TGT​CCA​T
• siRNA-3: ACA​CGA​TCC​TCA​CCG​AGA​A

### Western Blotting

Cells were harvested and total protein was extracted using radioimmunoprecipitation assay (RIPA) buffer (Fdbio science, Hangzhou, China) supplemented with phenylmethanesulfonyl fluoride (PMSF) (Fdbio science, Hangzhou, China), protease inhibitor (Fdbio science, Hangzhou, China), and phosphatase inhibitor (Fdbio science, Hangzhou, China). Protein concentration was determined by BCA protein quantitation assay (KeyGen Biotech, Nanjing, China). Thirty micrograms of protein from each sample was separated with 10% sodium dodecyl sulfate polyacrylamide gel electrophoresis (SDS-PAGE) and transferred onto polyvinylidene fluoride (PVDF) membranes (Roche, Switzerland). To prevent antibody binding to non-specific epitopes, blocking was performed with 5% bovine serum albumin (BSA) in Tris-buffered saline with 0.1% Tween 20 (TBST) for 1 h at room temperature. Then, the samples were incubated overnight at 4°C with the primary antibodies, including anti-SMG9 (1:1,000; Abcam, Cambridge, United Kingdom), anti-E-cadherin (1:1,000; Cell Signaling Technology, MA, United States), anti-Vimentin (1:1,000; Cell Signaling Technology, MA, United States), anti-N-cadherin (1:1,000; Cell Signaling Technology, MA, United States), anti-β-catenin (1:1,000; Cell Signaling Technology, MA, United States), anti-MMP2 (1:1,000; Abcam, Cambridge, United Kingdom), anti-MMP9 (1:1,000; Abcam, Cambridge, United Kingdom), anti-PCNA (1:1,000; Abcam, Cambridge, United Kingdom), anti-Bax (1:1,000; Abcam, Cambridge, United Kingdom), anti-Bcl-2 (1:1,000; Abcam, Cambridge, United Kingdom), and anti-GAPDH (1:1,000; Bioss, Beijing, China). The next day, membranes were washed with TBST and incubated with the suitable secondary antibody (1:5,000; Cell Signaling Technology, MA, United States). Samples were then washed three times with TBST for 5 min, and the target proteins on the membranes were visualized using Immobilon Western Chemiluminescent HRP Substrate (Millipore, MA, United States).

### CCK8 Assay

A CCK8 (Cell Counting Kit-8; Dojondo, Tabaru, Japan) assay was used to measure the cell proliferation. The cells were seeded in 96-well plates at a density of 1 × 10^4^ cells per well. The absorbance of wells at 450 nm (reference wavelength, 650 nm) was measured at 0, 24, 48, and 72 h after siRNA transfection with a microplate reader (BioTek EPOCH, United States). Each experiment was performed in triplicate, and the data were presented as mean ± standard deviation (SD).

### EdU Assay

Cell proliferation was detected by using BeyoClick™ EdU Cell Proliferation Kit with Alexa Fluor 594 (Beyotime, Shanghai, China) according to the manufacturer instructions. In brief, the SMG9 NC- or SMG9 siRNA-transfected SMMC-7721 and HepG2 cells were cultured in 96-well plates with 1 × 10^4^ cells/well, respectively. After 24 h of culture, the cells were labeled with 20 μM EdU for 2 h at 37°C, and then rinsed twice at room temperature in PBS. After labeling, the cells were fixed with 4% paraformaldehyde, permeabilized with 0.3% Triton X-100, incubated with 50 μl Click Additive Solution for 30 min protecting from light, and stained with 50 μl Hoechst 33,342 (Sigma-Aldrich, St. Louis, United States) for 15 min. Images were taken under a fluorescence microscopy (Leica, Germany) and the percentage of EdU-positive cells was calculated. The experiments were performed at least three times.

### Gene Set Enrichment Analysis

Gene expression data was obtained from TCGA database. To further identify pathways/gene sets associated with SMG9, the data was used to perform gene set enrichment analysis (GSEA). For GSEA, the expression of each gene on the rank list is weighted based on its Log FC. GSEA software (version: 4.1.0) was downloaded from broad institute (Subramanian et al., 2005) through the following link: http://software.broadinstitute.org/gsea/downloads.jsp.

### Cell Cycle Analysis

Cell cycle was analyzed using flow cytometry by measuring DNA content of cells with the Cell Cycle Detection Kit (KeyGen Biotech, Nanjing, China) assay. The experimental procedures were in line with the manufacturer’s instructions. SMMC-7721 and HepG2 cells in the logarithmic growth phase (1 × 10^5^/ml) were seeded in six-well plates. Then, SMG9 NC or SMG9 siRNA was transfected into SMMC-7721 and HepG2 cells with Lipofectamine™ 3,000. After 48 h in culture, the cells were harvested with 0.25% trypsin-EDTA (Invitrogen, Carlsbad, CA, United States) and centrifuged at 1,200 rpm for 5 min. Cells were then washed twice with cold PBS and fixed overnight in pre-cooled 70% ethanol at 4°C. The next day, ethanol was removed by centrifugation and cells were washed twice with cold PBS and incubated with 500 μl working solution of propidium iodide (PI)/RNase A at a volume ratio of 9:1 at 4°C for 30 min away from light. After that, the cell cycle was analyzed using BD FACS Calibur (BD Biosciences).

### Flow Cytometry

An Annexin V-FITC Apoptosis Kit (KeyGen Biotech, Nanjing, China) was used to analyze cell apoptosis according to the manufacturer’s instructions. SMMC-7721 and HepG2 cells of logarithmic growth phase (1 × 10^5^/ml) were seeded in six-well plates. Then, SMG9 NC or SMG9 siRNA was transfected into SMMC-7721 and HepG2 cells with Lipofectamine™ 3,000. Forty eight hours post-transfection, all cells (including cells in the supernatant) were harvested with *trypsin without EDTA* (Beyotime, Shanghai, China) and centrifuged at 1,200 rpm for 5 min. After washing with cold PBS twice, the cells were resuspended in 500 μl binding buffer and incubated with 5 μl Annexin V- FITC and 5 μl PI *at room temperature for 15* min *in the dark.* Then, the samples were analyzed on a flow cytometer (BD FACS Calibur System; BD, United States).

### Scratch Wound Healing Assay

The SMG9 NC-or SMG9 siRNA-transfected SMMC-7721 and HepG2 cells were placed in six-well plates at a density of 1 × 10^6^/ml respectively and incubated at 37 °C with 5% (v/v) CO_2_ until they reached 100% confluence. The monolayer was scratched with a sterile 10 µl pipette tip. After washing away cell fragments, the cells were cultured with serum-free DMEM and photographed under a light microscope (Leica, Germany) at 0 h (H0), 24, 48 and 72 h (H) after scratch was made. The distance between the two edges of the scratch was measured and data were presented as migration rate. The formula used for the calculation of the cell migration rate was as follows: migration rate (%) = (1-H cell migration distance/H0 cell migration distance) × 100%.

### Transwell Migration Assay

Cell migration was determined by transwell chambers (Corning, NY, United States) migration assay. The SMG9 NC- or SMG9 siRNA-transfected SMMC-7721 and HepG2 cells (1 × 10^5^ cells) were plated in the upper chamber in 200 uL serum-free DMEM. The bottom chamber of the transwell system was filled with 500 uL DMEM containing 20% FBS. After incubation for 24 h, the non-migrating cells on the upper side of the membrane were carefully removed using a cotton swab, while the migrated cells attached to the bottom side of the membrane were fixed with 4% paraformaldehyde for 30 min and colored with 0.1% crystal violet. The stained cells were then washed 2–3 times (5 min each time) with PBS, visualized and counted in five random fields under the microscope (Leica, Germany). All experiments were repeated three times, independently.

### Transwell Invasion Assay

Cell invasion was evaluated by transwell assay using BD BioCoat Matrigel invasion chambers (BD Biosciences, MA, United States). Transwell chambers were placed in a sterile 24-well culture plate initially. The SMG9 NC- or SMG9 siRNA-transfected SMMC-7721 and HepG2 cells were seeded in the upper chamber in 200 uL serum-free DMEM at a density of 1 × 10^5^/ml. At the same time, 500 µl of DMEM supplemented with 20% FBS was added to the bottom chamber. After 48 h of incubation in a 5% (v/v) CO_2_ incubator at 37°C, the matrigel and noninvasive cells on the upper surface of the filters were removed, and then the filters were fixed with 70% methanol and stained with 0.1% crystal violet. Finally, the number of invasive cells was quantified by counting five randomly selected fields under the microscope (Leica, Germany). The experiments were carried out three times independently and data were presented as mean ± SD.

### Statistical Analysis

Statistical analyses were conducted using SPSS software version 23.0 (IBM, Armonk, New York). Depending on the character of the data, Students t test was used for intergroup comparisons of numerical variables, and Fisher’s exact test was used for comparisons of categorical variables. The influence of different prognostic elements on OS and DFS of HCC patients was evaluated by Kaplan-Meier analysis and Cox regression. Two-tailed *p* values <0.05 were considered as statistically significant.

## Results

### SMG9 Is Upregulated in Human Hepatocellular Carcinoma Tissues Compared With Adjacent Non Cancerous Liver Tissues

**FIGURE 1:**
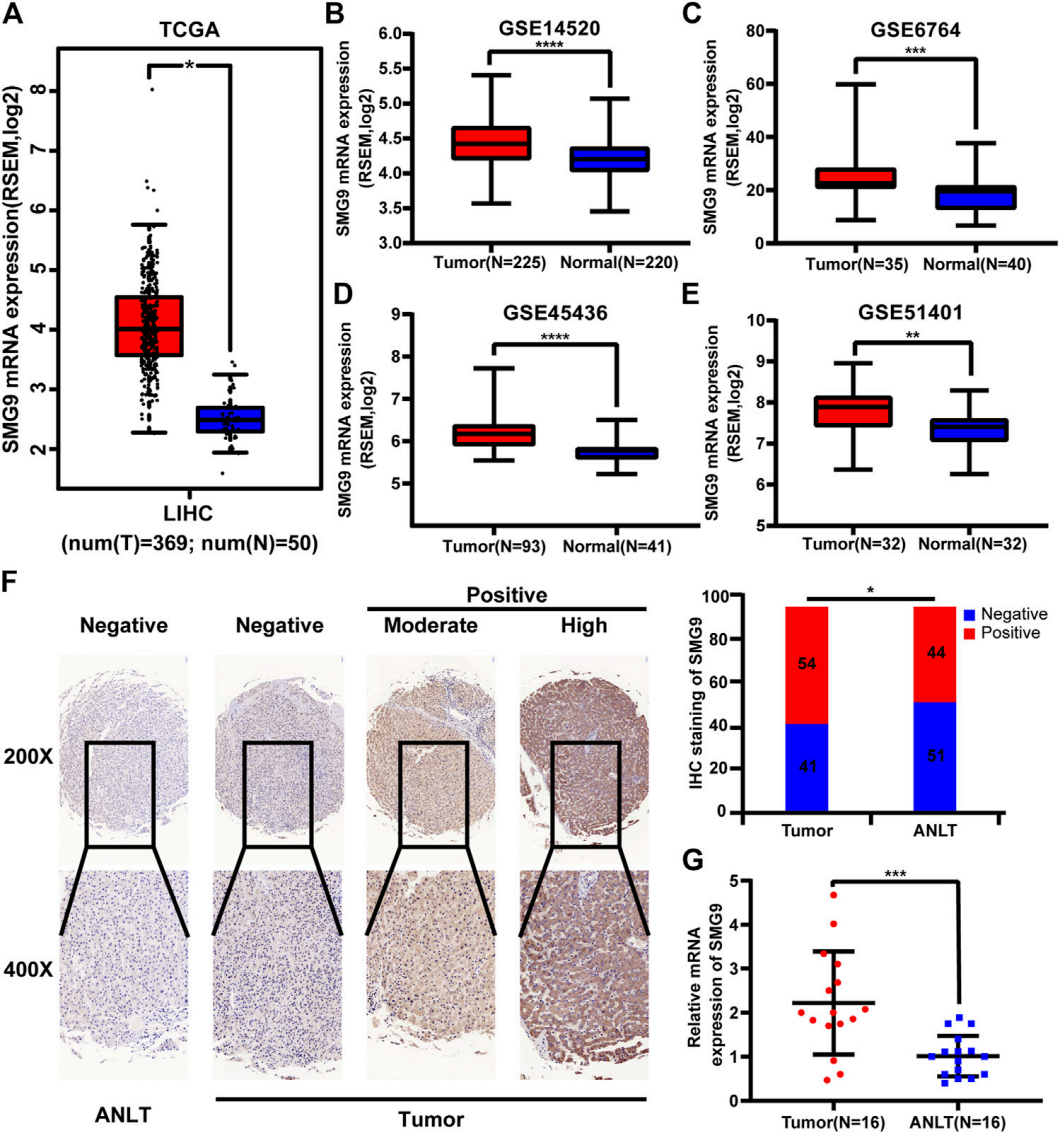
SMG9 is upregulated in HCC tissues compared with ANLTs. SMG9 was highly expressed in HCC tissues compared to normal tissues according to analysis of data from TCGA (**A**; tumor, *n* = 369; normal, *n* = 50) and GEO (**B–E**; GSE14520, tumor, *n* = 225; normal, *n* = 220; GSE6764, tumor, *n* = 35; normal, *n* = 40; GSE45436, tumor, *n* = 93; normal, *n* = 41; GSE51401, tumor, *n* = 32; normal, *n* = 32). **(F)** The IHC staining of SMG9 in human HCC tissues and ANLTs. **(G)** RT-qPCR analysis of SMG9 expression in 16 pairs of HCC tissues and corresponding ANLTs. All: ^*^ *p* < 0.05, **p < 0.01, ***p < 0.001, ****p < 0.0001.

Data from TCGA and GEO (GSE14520, GSE6764, GSE45436, and GSE51401 datasets) were used for gene expression analysis. SMG9 expression was found to be significantly increased in the HCC tissues compared with that in the corresponding normal tissues, and the findings are shown in Figures 1A–E. IHC staining of 95-paired tissues showed that SMG9 expression was significantly higher in HCC tissues than in the ANLTs (Figure 1F). In addition, the mRNA expression of SMG9 was confirmed by RT-qPCR, showing that SMG9 was upregulated in 16 sets of fresh HCC tissues in contrast with ANLTs (Figure 1G). The relationships between SMG9 expression and clinical characteristics of HCC patients were showed in Table 1. The expression level of SMG9 was significantly associated with tumor number (*p* = 0.014), tumor size (*p* = 0.015), and TNM stage (*p* = 0.025). These findings indicate that SMG9 is upregulated in human HCC tissues and is correlated with worse clinicopathology features of HCC patients.

### SMG9 Expression Is Correlated With Poor Prognosis in Human Hepatocellular Carcinoma

**FIGURE 2:**
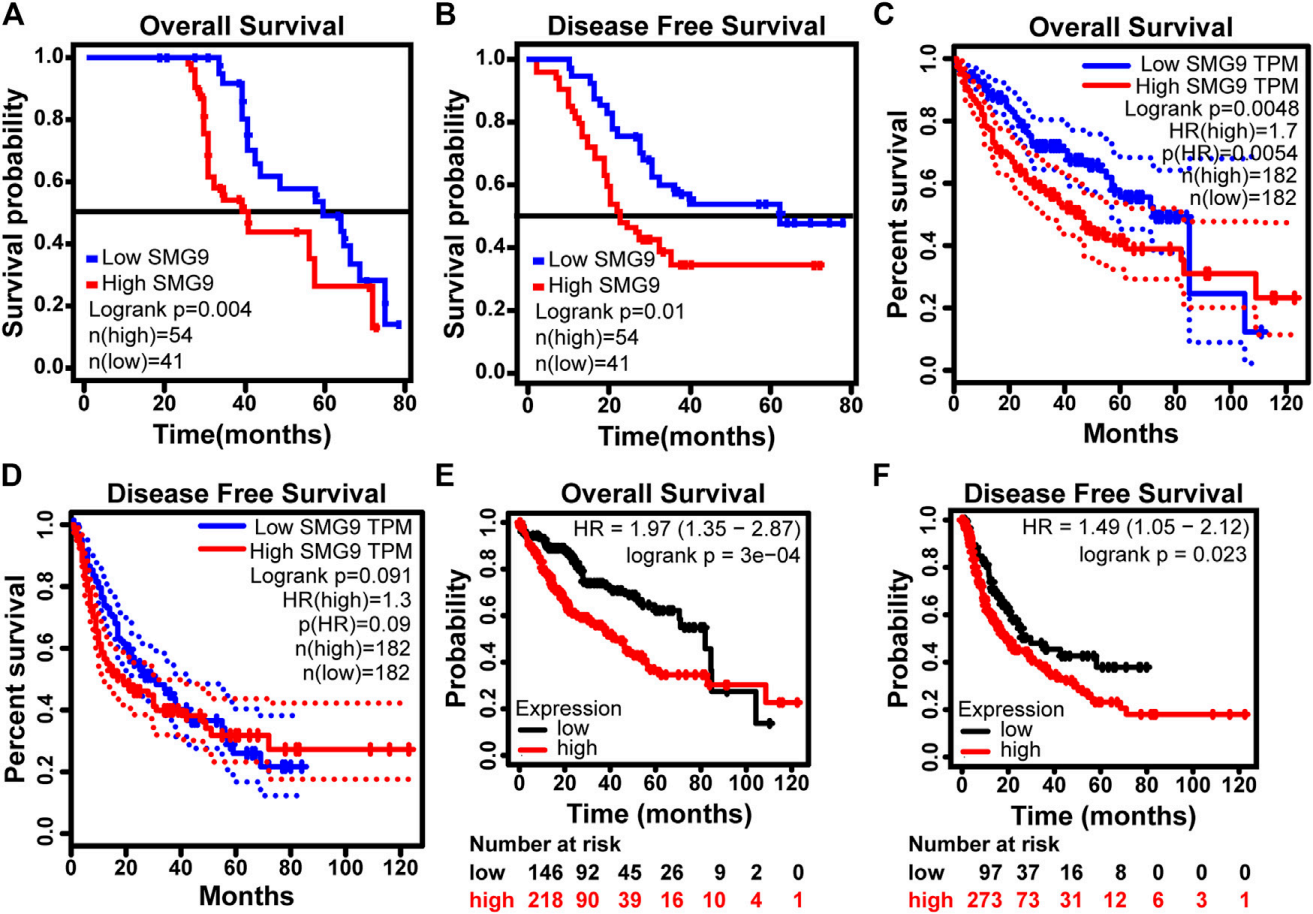
SMG9 expression is correlated with poor prognosis in human HCC. OS **(A)** and DFS **(B)** curves in 95 patients with HCC stratified by SMG9 expression. OS **(C)** and DFS **(D)** analysis of TCGA. Kaplan-Meier survival curves of OS **(E)** and DFS **(F)** generated from Kaplan*-*Meier plotter for high- and low- SMG9 expression groups.

To determine the prognostic value of SMG9, Kaplan–Meier survival analysis was carried out for 95 HCC cases, the results are shown in Figures 2A,B. High SMG9 expression was associated with poor OS and DFS. Cox proportional hazards analysis for 364 HCC cases from the TCGA database showed that patients with higher SMG9 expression had poorer prognosis (Figures 2C,D). To further validate the results, Kaplan-Meier survival curves of OS and DFS generated from Kaplan*-*Meier plotter (Nagy et al., 2021) for high- and low-SMG9 expression groups also showed the same results (Figures 2E,F). Multivariate analysis showed that SMG9 expression was an independent prognostic factor for OS [hazard ratio (HR) = 3.326, 95% confidence interval (CI): 1.644–6.731, *p* = 0.001] (Table 2) and DFS (HR = 3.661, 95% CI: 1.797–7.461, *p* < 0.001) (Table 3). In general, the results suggest that high SMG9 expression is correlated with poor prognosis in human HCC.

**TABLE 2:** Univariate and multivariate Cox regression analysis of risk factors associated with OS.

Variables	Univariate analysis			Multivariate analysis		
	HR	95% CI	*p* value	HR	95% CI	*p* value
Sex	1.252	0.697–2.250	0.452			
Age	1.313	0.734–2.349	0.359			
TNM stage	1.960	1.088–3.533	**0.025**	1.361	0.710–2.608	0.353
Tumor thrombus	1.550	0.828–2.901	0.171			
HBV	1.316	0.722–2.396	0.370			
Tumor number	0.547	0.277–1.081	0.082	0.564	0.265–1.202	0.138
Tumor size (≥5 cm)	0.534	0.290–0.983	**0.044**	0.426	0.218–0.832	**0.012**
AFP	2.610	1.389–4.903	**0.003**	2.288	1.194–4.387	**0.013**
Pathological grading	1.005	0.541–1.869	0.983			
SMG9	2.401	1.292–4.460	**0.006**	3.326	1.644–6.731	**0.001**

Bold values indicate statistical significance. If the *p* value was <0.05, the data were considered to be statistically significant difference.

**TABLE 3:** Univariate and multivariate Cox regression analysis of risk factors associated with DFS.

Variables	Univariate analysis			Multivariate analysis		
	HR	95% CI	*p* value	HR	95% CI	*p* value
Sex	1.313	0.732–2.356	0.361			
Age	1.403	0.783–2.516	0.255			
TNM stage	2.044	1.134–3.684	**0.017**	1.532	0.800–2.935	0.198
Tumor thrombus	1.430	0.766–2.668	0.261			
HBV	1.227	0.673–2.236	0.504			
Tumor number	0.526	0.260–1.063	0.074	0.534	0.257–1.111	0.093
Tumor size (≥ 5cm)	0.535	0.291–0.985	**0.045**	0.387	0.197–0.760	**0.006**
AFP	2.890	1.540–5.423	**0.001**	2.326	1.222–4.425	**0.010**
Pathological grading	0.956	0.514–1.780	0.888			
SMG9	2.645	1.404–4.983	**0.003**	3.661	1.797–7.461	**<0.001**

Bold values indicate statistical significance. If the *p* value was <0.05, the data were considered to be statistically significant difference.

### SMG9 Promotes Proliferation of Hepatocellular Carcinoma Cells

**FIGURE 3:**
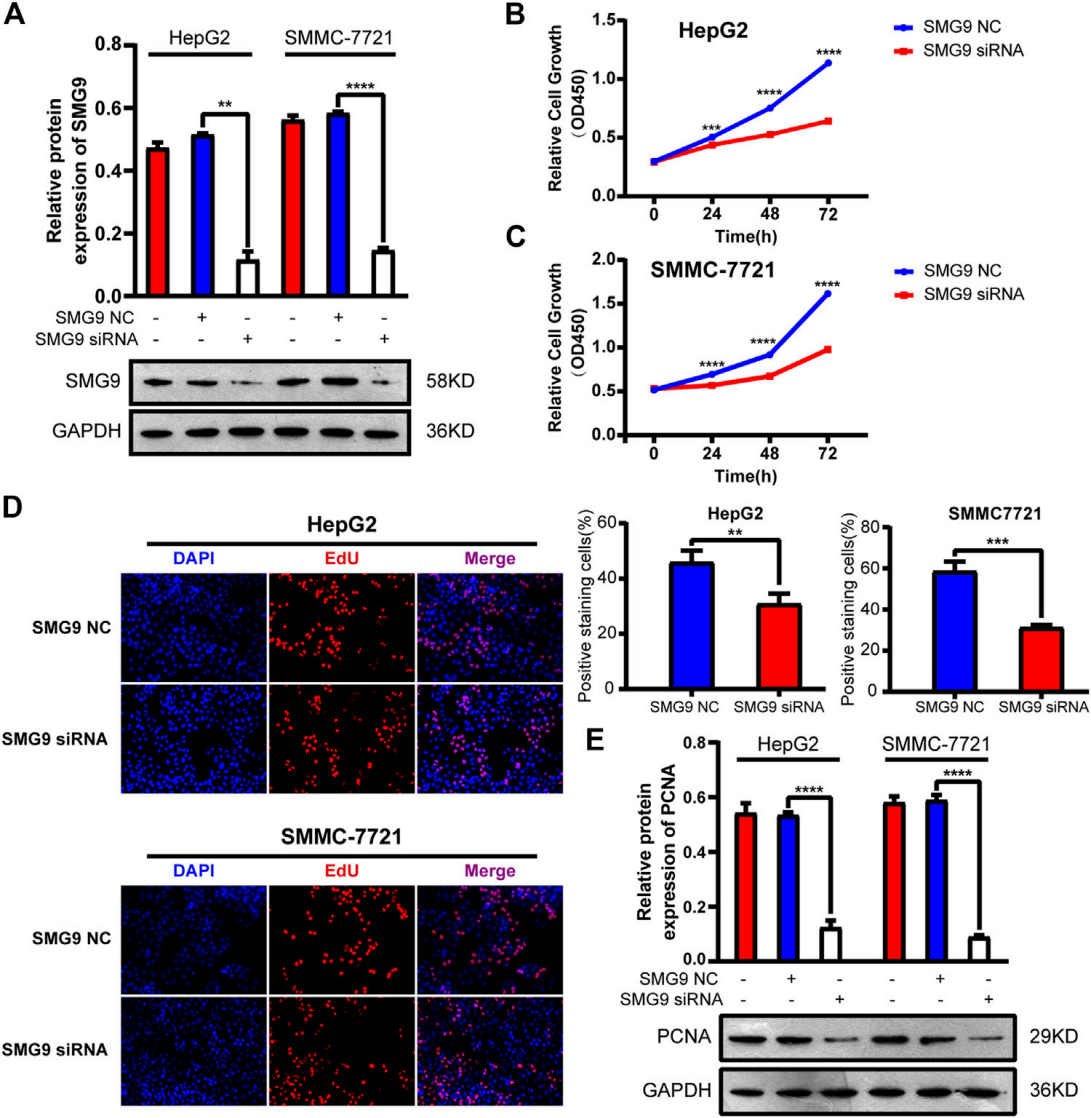
Silence of SMG9 attenuates HCC cell proliferation**. (A)** Knockdown efficiency of SMG9 siRNA was determined by western blotting. **(B,C)** Effects of SMG9 on cell proliferation was evaluated by CCK8 assay in siRNA-transfected SMMC-7721 and HepG2 cells. **(D)** Cell proliferation was determined by the EdU assay in SMMC-7721 and HepG2 cells transfected with SMG9 NC or SMG9 siRNA. Images were captured on an inverted fluorescence microscope. **(E)** The protein expression level of PCNA was measured by western blotting analysis in SMMC-7721 and HepG2 cells transfected with SMG9 NC or SMG9 siRNA. Data were presented as the mean ± SD of three independent repeats of the experiments. **p < 0.01, ***p < 0.001, ****p < 0.0001.

To explore the function of SMG9 in HCC, we transfected SMMC-7721 and HepG2 cell lines with three siRNAs (siRNA-1, siRNA-2, and siRNA-3) targeting the coding sequence of SMG9 and negative control (NC) siRNA. Western blotting showed that protein expression of SMG9 was markedly decreased in SMG9 siRNA-transfected cells compared with that in control cells (Figure 3A). CCK8 assay was applied to investigate the effect of SMG9 on cell proliferation. We observed that cell proliferation was significantly inhibited in both SMMC-7721 and HepG2 cell lines at 24, 48 and 72 h after SMG9-siRNA transfection as compared to the control group (Figures 3B,C). Next, the function of SMG9 on HCC cell proliferation was detected by EdU staining. The results showed that the proliferation of SMMC-7721 and HepG2 cells was significantly reduced in SMG9 siRNA group as compared to SMG9 NC group (Figure 3D). Furthermore, we found that PCNA (cell proliferation marker) expression was significantly reduced after SMG9 siRNA transfection (Figure 3E). Collectively, these data indicate that inhibition of SMG9 attenuates HCC cell proliferation.

### Knockdown of SMG9 Induces Cell Cycle Arrest and Apoptosis in Hepatocellular Carcinoma Cells

**FIGURE 4:**
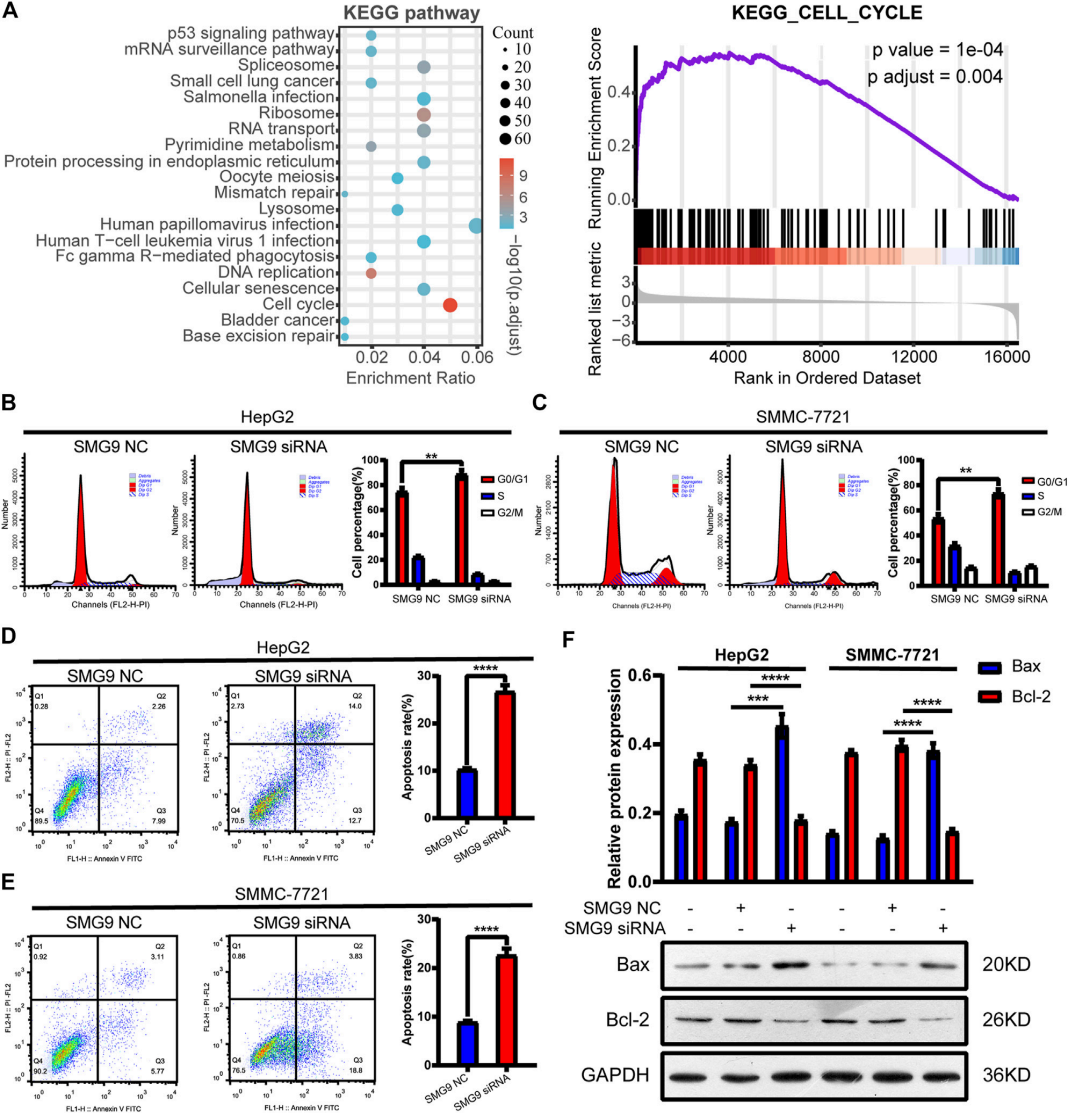
SMG9 siRNA induces G0/G1 phase cell cycle arrest and apoptosis in HCC cells. **(A)** GSEA analysis showed that cell cycle was significantly associated with SMG9 in HCC based on TCGA datasets. **(B,C)** Knockdown of SMG9 arrested cell cycle at G0/G1 phase in SMMC-7721 and HepG2 cells. **(D,E)** Cell apoptosis of SMMC-7721 and HepG2 cells transfected with siRNA NC or siRNA SMG9 was measured by flow cytometry. **(F)** Bax and Bcl-2 protein expression levels were measured by Western blotting in SMMC-7721 and HepG2 cells transfected with siRNA NC or siRNA SMG9. Data were presented as the mean ± SD of three independent repeats of the experiments. **p < 0.01, ***p < 0.001, ****p < 0.0001.

Since cell cycle and apoptosis are tightly associated with cell proliferation, we then determine whether SMG9 could affect cell cycle and apoptosis in HCC cells (Teng et al., 2008; Zheng et al., 2019). To understand whether SMG9 affected the cell cycle progression, GSEA was performed using high-throughput RNA-sequencing data of the TCGA database. As shown in Figure 4A, cell cycle was significantly associated with SMG9 in HCC. Then, flow cytometry was used to assess the effect of SMG9 on cell cycle. SMG9 siRNA transfection markedly increased the percentage of cells in the G0/G1 phase and decreased the percentage of cells in the S phase of HepG2 and SMMC-7721 cells as compared to the NC group (Figures 4B,C). *Meanwhile*, *the role of* SMG9 *in* regulating cell *apoptosis* was also evaluated by flow cytometry, and the results showed that knockdown of SMG9 increased the percentage of apoptosis cells in HepG2 and SMMC-7721 cells (Figures 4D,E). Additionally, western blotting results showed that Bax protein level was increased and Bcl-2 protein level was decreased in SMMC-7721 and HepG2 cells after knockdown of SMG9 (Figure 4F). Taken together, our data reveal that SMG9 siRNA induces G0/G1 phase cell cycle arrest and cell apoptosis in HCC cells.

### SMG9 Promotes Migration and Invasion of SMMC-7721 and HepG2 Cells

**FIGURE 5:**
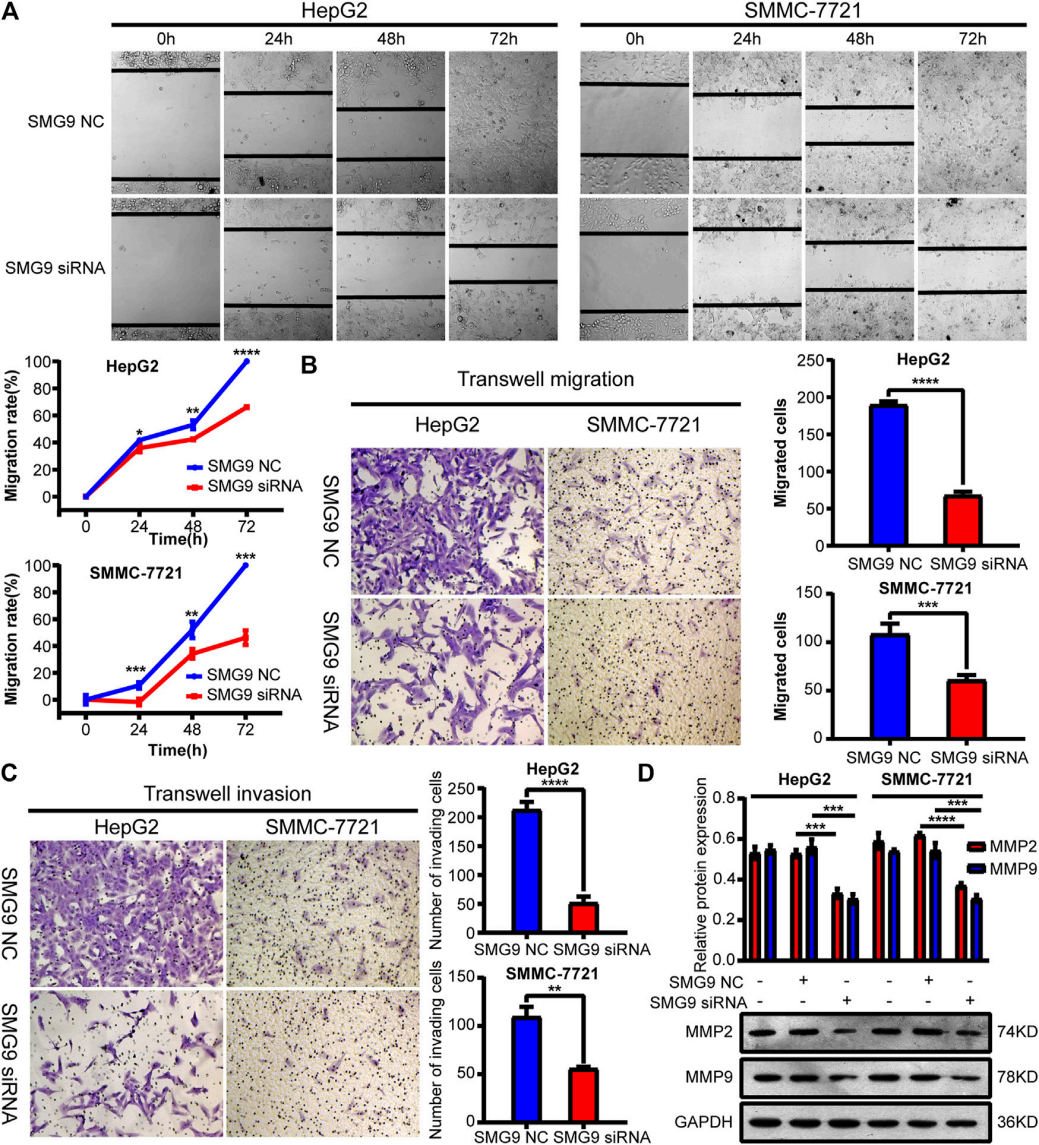
Downregulation of SMG9 represses migration and invasion of SMMC-7721 and HepG2 cells. **(A,B)** Scratch wound-healing assay and transwell migration assay were applied to determine cell migratory capability of SMMC-7721 and HepG2 cells transfected with SMG9 NC or SMG9 siRNA. **(C)** Transwell invasion assay was conducted to assess the effects of SMG9 knockdown on the invasive potential of SMMC-7721 and HepG2 cells. **(D)** Levels of MMP2 and MMP9 were measured by western blotting in SMMC-7721 and HepG2 cells transfected with SMG9 NC or SMG9 siRNA, respectively. Data were presented as the mean ± SD of three independent repeats of the experiments. ^*^ *p* < 0.05, **p < 0.01, ***p < 0.001, ****p < 0.0001.

To determine the effect of SMG9 on cell motility, scratch wound healing assay along with transwell migration assay were employed. As a result, cell migration capacity was significantly decreased by treatment with SMG9 siRNA in both SMMC-7721 and HepG2 cells compared with control cells at 24, 48 and 72 h (Figures 5A,B). In the transwell invasion experiments, downregulation of SMG9 significantly reduced the invasive capacity of both SMMC-7721 and HepG2 cells (Figure 5C). In addition, MMP2 and MMP9 protein expression levels were assessed by western blotting, as both MMP2 and MMP9 play key roles in regulating cell migration (Muscella et al., 2020). We observed that both MMP2 and MMP9 expression were significantly down-regulated after transfecting with SMG9 siRNA in SMMC-7721 and HepG2 cells (Figure 5D). Thus, these results suggest that downregulation of SMG9 represses migration and invasion of SMMC-7721 and HepG2 cells.

### SMG9 Facilitates the EMT Process and Activates Wnt/β-Catenin Signaling Pathway in Hepatocellular Carcinoma Cells

**FIGURE 6:**
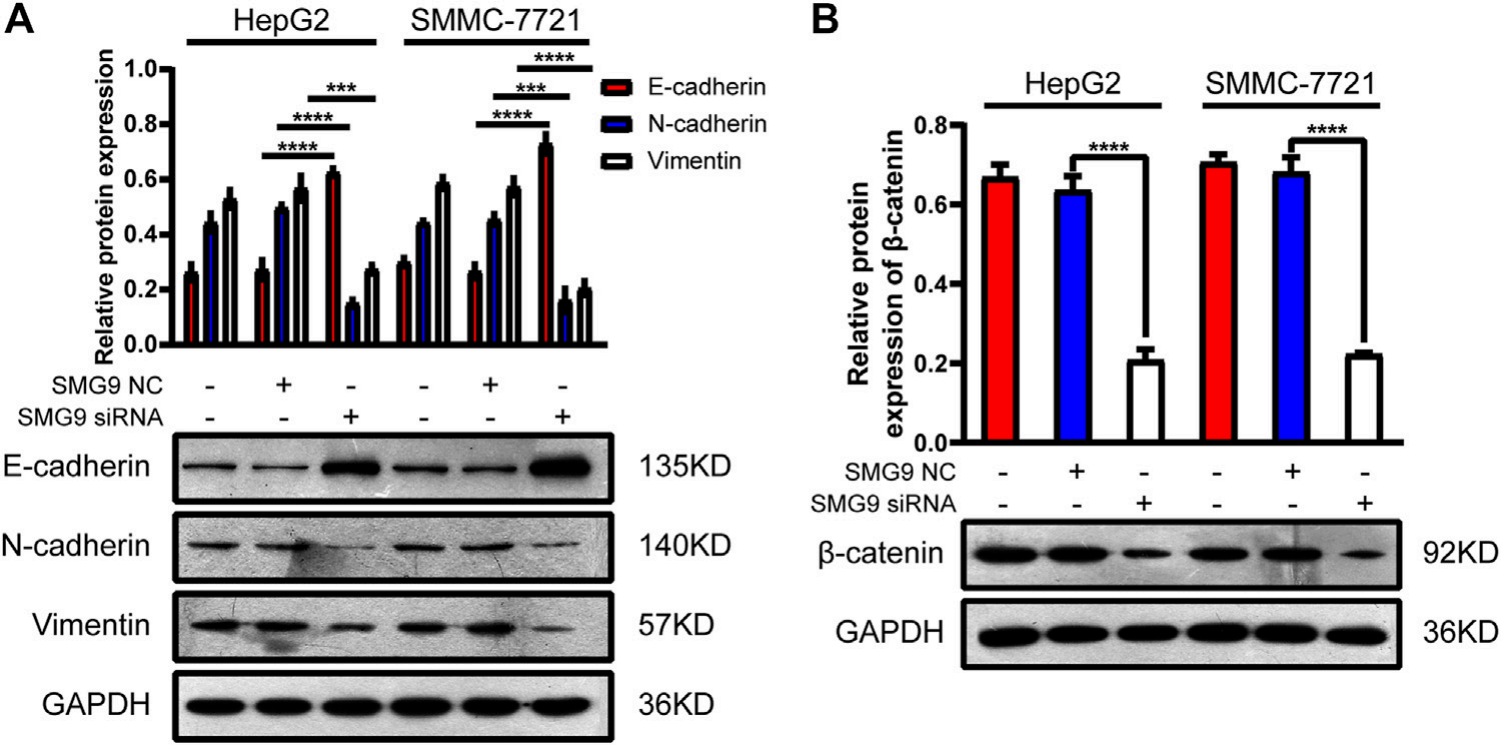
SMG9 promotes the process of EMT and activates Wnt/β-catenin signaling pathway in HCC cells. **(A)** The protein expression levels of E-cadherin, N-cadherin, and Vimentin were measured by western blotting in SMMC-7721 and HepG2 cells transfected with SMG9 NC or SMG9 siRNA, respectively. **(B)** The level of β-catenin protein was detected by western blotting in SMMC-7721 and HepG2 cells transfected with SMG9 NC or SMG9 siRNA. Data were presented as the mean ± SD of three independent repeats of the experiments. ***p < 0.001, ****p < 0.0001.

EMT has been recognized as an important step toward high invasion and metastasis of many cancers including HCC (Jia et al., 2021). To investigate the role of SMG9 in EMT, we knocked down SMG9 in SMMC-7721 and HepG2 cells using siRNA specific SMG9, and the relative proteins of EMT were detected by western blotting. The results showed that the expression level of E-cadherin was significantly increased, however, the expression levels of vimentin and N-cadherin were significantly reduced upon SMG9 knockdown (Figure 6A). The activation of Wnt/β-catenin signaling pathway is closely related to tumor progression including proliferation, apoptosis, invasion, and migration (Li et al., 2021). To investigate whether Wnt/β-catenin was implicated in the progression of HCC, β-catenin protein level was detected in SMG9 NC or SMG9 siRNA-transfected SMMC-7721 and HepG2 cells. SMG9 siRNA markedly reduced the expression level of β-catenin as compared to the control group (Figure 6B). In summary, these findings demonstrate that SMG9 promotes the process of EMT and activates Wnt/β-catenin signaling pathway in HCC cells.

## Discussion

In this study, we have investigated the expression level of SMG9 in HCC tissues and assessed the prognostic role of this gene for survival of HCC patients. We have identified that knockdown of SMG9 contributes to reduced cell proliferation, enhanced apoptosis, and decreased migration and invasion abilities. In addition, we have further demonstrated that EMT and the Wnt/β-catenin signaling pathway are involved in SMG9-mediated HCC progression.

According to *GLOBOCAN* 2020 (http://gco.iarc.fr/), HCC, with high incidence and mortality, is one of the most common malignant tumors in the world, especially in China. GEO and TCGA are public genomics data repositories of next-generation sequencing and high-throughput microarrays for many tumor types and tumor tissues. Here, microarray data from GEO and RNA sequencing data from TCGA were analyzed to investigate *the relationship between* SMG9 *and HCC*. *Analysis* revealed that the mRNA expression level of SMG9 was upregulated in HCC tissues than in normal tissues. Using RT-qPCR, we also found SMG9 expression was significantly higher in 16 sets of fresh HCC tissues than in ANLTs, in line with our gene analysis findings. Furthermore, the prognostic value of SMG9 was investigated. TCGA database-survival analysis showed that HCC patients with high SMG9 expression had significantly worse prognosis than those with low SMG9 expression. Consistent with the *TCGA* results, our studies using IHC staining, Kaplan-Meier analysis and Cox regression also showed that SMG9 protein expression in the histological specimens was strongly linked with clinicopathological characteristics and survival time of HCC patients. Collectively, all these results suggest that SMG9 is closely related to HCC and plays an important role in the progression of HCC.

SMG9 has been first defined as a participant in NMD since 2009, which directly binds SMG1 and SMG8, forming a stable SMG1–SMG8–SMG9 complex (Yamashita et al., 2009). As an essential component of the NMD, SMG9 plays a critical role in the NMD pathway (Yamashita et al., 2009; Fernandez et al., 2011; Zhu et al., 2019). Growing evidences have shown that NMD pathway is implicated in many biological processes including embryonic development, cell differentiation, stress responses and immune response (Nasif et al., 2018). Disruption of the NMD can lead to a plethora of human genetic diseases, including cancer (Lindeboom et al., 2019). Notably, NMD exerts dual effects in tumor progression (Nogueira et al., 2021). On one hand, tumors have exploited NMD to downregulate key tumor-suppressor gene expression, whereas on the other hand, adjusted NMD activity to adapt to their microenvironment (Popp and Maquat, 2018). NMD targets and regulates a wide variety of transcripts that are important for tumorigenesis, including proteins involved in cell cycle, cell growth, apoptosis, growth factor signaling, and cell migration (Wang et al., 2011). In this study, we have identified the role of SMG9, the key player in NMD, in the progression of HCC. Since sustaining proliferative signaling, resisting cell death, and activating invasion/metastasis are the basic hallmarks of cancer (Hanahan and Weinberg, 2011), we have analyzed the alteration of cell proliferation, apoptosis, migration and invasion by *in vitro* studies. First, CCK8 assay and EdU staining were used to explore the function of SMG9 on HCC cell proliferation. These results showed that SMG9 siRNA inhibit HCC cell proliferation. Subsequently, analysis of cell cycle phase distribution and apoptosis confirmed SMG9 siRNA induced cell cycle arrest at G0/G1 phase and apoptosis in HCC cells. Furthermore, *migration and invasion assay* showed that SMG9 promoted HCC cell migration and invasion. Taking together, all the above results indicate that SMG9 promotes HCC progression by promoting proliferation, cell cycle progression, apoptosis resistance, migration and invasion in HCC cells.

Mechanically, EMT is tightly related to not only invasion and metastasis but also proliferation (Chen et al., 2020 *).* Meanwhile, there is a strong connection between EMT and HCC (Tong et al., 2020). The Wnt/β-catenin signaling pathway is involved in the development of various human cancers and one of the most well-characterized oncogenic pathway in *HCC* (Hoshida et al., 2009; Huang et al., 2019; Bi et al., 2020; Wang et al., 2020 *). A* variety of key cellular processes including proliferation, apoptosis, migration and invasion are involved in the progression of HCC in a Wnt/β-catenin-dependent manner (Huang et al., 2020; Lin et al., 2020). Thus, the Wnt/β-catenin signaling pathway and EMT have potential value in the diagnosis and prognosis of HCC. In our study, SMG9 siRNA decreased the expression levels of Vimentin, N-cadherin, and β-catenin whereas increased the protein expression level of E-cadherin, indicating that SMG9 mediates HCC cell proliferation, apoptosis, migration and invasion via activating EMT and the Wnt/β-catenin signaling pathway.

In summary, our findings show that higher expression of SMG9 is predictive of a poorer prognosis in HCC patients and plays a crucial role in HCC progression via EMT and the Wnt/β-catenin signaling pathway. The molecular mechanisms of SMG9 promoting HCC cell proliferation, apoptosis, migration and invasion remain to be further elucidated in the future study. *Recently*, some researchers have *reported that* SMG9 positively regulates ferroptosis independent of its activity in NMD, implicating that SMG9 may has biological functions beyond NMD (Fernandez et al., 2011; Han et al., 2021). Accordingly, understanding the other potential functions of SMG9 during HCC *progression* is an attractive direction for future work, which will provide a new strategy for liver cancer prevention and treatment.

## Data Availability

The raw data supporting the conclusions of this article will be made available by the authors, without undue reservation.

## Abbreviations

AFP, alpha-fetoprotein; ANLTs, adjacent non-cancerous liver tissues; CAFs, cancer-associated fibroblasts; DCP, des-gamma-carboxy prothrombin; DFS, disease free survival; EMT, epithelial-mesenchymal transformation; GEO, gene expression omnibus; GSEA, gene set enrichment analysis; IHC, immunohistochemistry; NMD, nonsense-mediated decay; OS, overall survival; PTCs, Premature translation termination codons; RT-qPCR, Reverse transcription-quantitative polymerase chain reaction; siRNA, small interfering RNA; SMG1C, SMG1 complex; TCGA, the cancer genome atlas; TME, tumor microenvironment.

## References

[B1] Alzahrani F. Kuwahara H. Long Y. Al-Owain M. Tohary M. AlSayed M. (2020). Recessive, Deleterious Variants in SMG8 Expand the Role of Nonsense-Mediated Decay in Developmental Disorders in Humans. Am. J. Hum. Genet. 107, 1178–1185. 10.1016/j.ajhg.2020.11.007 33242396 PMC7820624

[B2] Arnold M. Abnet C. C. Neale R. E. Vignat J. Giovannucci E. L. McGlynn K. A. (2020). Global Burden of 5 Major Types of Gastrointestinal Cancer. Gastroenterology 159, 335–349. 10.1053/j.gastro.2020.02.068 32247694 PMC8630546

[B3] Bi Z. Li Q. Dinglin X. Xu Y. You K. Hong H. (2020). Nanoparticles (NPs)‐Meditated LncRNA AFAP1‐AS1 Silencing to Block Wnt/ β ‐Catenin Signaling Pathway for Synergistic Reversal of Radioresistance and Effective Cancer Radiotherapy. Adv. Sci. 7, 2000915. 10.1002/advs.202000915 PMC7509644 32999837

[B4] Bray F. Ferlay J. Soerjomataram I. Siegel R. L. Torre L. A. Jemal A. (2018). Global Cancer Statistics 2018: GLOBOCAN Estimates of Incidence and Mortality Worldwide for 36 Cancers in 185 Countries. CA: A Cancer J. Clinicians 68, 394–424. 10.3322/caac.21492 30207593

[B5] Chen S. Shen J. Zhao J. Wang J. Shan T. Li J. (2020). Magnolol Suppresses Pancreatic Cancer Development *In Vivo* and *In Vitro* via Negatively Regulating TGF-β/Smad Signaling. Front. Oncol. 10, 597672. 10.3389/fonc.2020.597672 33344246 PMC7738609

[B6] Deniaud A. Karuppasamy M. Bock T. Masiulis S. Huard K. Garzoni F. (2015). A Network of SMG-8, SMG-9 and SMG-1 C-Terminal Insertion Domain Regulates UPF1 Substrate Recruitment and Phosphorylation. Nucleic Acids Res. 43, 7600–7611. 10.1093/nar/gkv668 26130714 PMC4551919

[B7] Ding X. He M. Chan A. W. H. Song Q. X. Sze S. C. Chen H. (2019). Genomic and Epigenomic Features of Primary and Recurrent Hepatocellular Carcinomas. Gastroenterology 157, 1630–1645. 10.1053/j.gastro.2019.09.005 31560893

[B8] Fernández I. S. Yamashita A. Arias-Palomo E. Bamba Y. Bartolomé R. A. Canales M. A. (2011). Characterization of SMG-9, an Essential Component of the Nonsense-Mediated mRNA Decay SMG1C Complex. Nucleic Acids Res. 39, 347–358. 10.1093/nar/gkq749 20817927 PMC3017601

[B9] Forner A. Reig M. Bruix J. (2018). Hepatocellular Carcinoma. The Lancet 391, 1301–1314. 10.1016/S0140-6736(18)30010-2 29307467

[B10] Giannelli G. Koudelkova P. Dituri F. Mikulits W. (2016). Role of Epithelial to Mesenchymal Transition in Hepatocellular Carcinoma. J. Hepatol. 65, 798–808. 10.1016/j.jhep.2016.05.007 27212245

[B11] Han L. Bai L. Fang X. Liu J. Kang R. Zhou D. (2021). SMG9 Drives Ferroptosis by Directly Inhibiting GPX4 Degradation. Biochem. Biophysical Res. Commun. 567, 92–98. 10.1016/j.bbrc.2021.06.038 34146907

[B12] Hanahan D. Weinberg R. A. (2011). Hallmarks of Cancer: the Next Generation. Cell 144, 646–674. 10.1016/j.cell.2011.02.013 21376230

[B13] Hernandez-Gea V. Toffanin S. Friedman S. L. Llovet J. M. (2013). Role of the Microenvironment in the Pathogenesis and Treatment of Hepatocellular Carcinoma. Gastroenterology 144, 512–527. 10.1053/j.gastro.2013.01.002 23313965 PMC3578068

[B14] Hoshida Y. Nijman S. M. B. Kobayashi M. Chan J. A. Brunet J.-P. Chiang D. Y. (2009). Integrative Transcriptome Analysis Reveals Common Molecular Subclasses of Human Hepatocellular Carcinoma. Cancer Res. 69, 7385–7392. 10.1158/0008-5472.CAN-09-1089 19723656 PMC3549578

[B15] Hou Y. Wang Z. Huang S. Sun C. Zhao J. Shi J. (2019). SKA3 Promotes Tumor Growth by Regulating CDK2/P53 Phosphorylation in Hepatocellular Carcinoma. Cell Death Dis 10, 929. 10.1038/s41419-019-2163-3 31804459 PMC6895034

[B16] Huang G. Liang M. Liu H. Huang J. Li P. Wang C. (2020). CircRNA hsa_circRNA_104348 Promotes Hepatocellular Carcinoma Progression through Modulating miR-187-3p/RTKN2 axis and Activating Wnt/β-Catenin Pathway. Cel Death Dis 11, 1065. 10.1038/s41419-020-03276-1 PMC7734058 33311442

[B17] Huang L. Luo E.-L. Xie J. Gan R.-H. Ding L.-C. Su B.-H. (2019). FZD2 Regulates Cell Proliferation and Invasion in Tongue Squamous Cell Carcinoma. Int. J. Biol. Sci. 15, 2330–2339. 10.7150/ijbs.33881 31595151 PMC6775310

[B18] Jia L. Li J. Li P. Liu D. Li J. Shen J. (2021). Site-specific Glycoproteomic Analysis Revealing Increased Core-Fucosylation on FOLR1 Enhances Folate Uptake Capacity of HCC Cells to Promote EMT. Theranostics 11, 6905–6921. 10.7150/thno.56882 34093861 PMC8171077

[B19] Kabashima A. Shimada S. Shimokawa M. Akiyama Y. Tanabe M. Tanaka S. (2021). Molecular and Immunological Paradigms of Hepatocellular Carcinoma: Special Reference to Therapeutic Approaches. J. Hepatobiliary Pancreat. Sci. 28, 62–75. 10.1002/jhbp.874 33259135

[B20] Kanwal F. Singal A. G. (2019). Surveillance for Hepatocellular Carcinoma: Current Best Practice and Future Direction. Gastroenterology 157, 54–64. 10.1053/j.gastro.2019.02.049 30986389 PMC6636644

[B21] Lecoquierre F. Bonnevalle A. Chadie A. Gayet C. Dumant‐Forest C. Renaux‐Petel M. (2019). Confirmation and Further Delineation of the SMG9‐deficiency Syndrome, a Rare and Severe Developmental Disorder. Am. J. Med. Genet. 179, 2257–2262. 10.1002/ajmg.a.61317 31390136

[B22] Li L.-y. Yang J.-f. Rong F. Luo Z.-p. Hu S. Fang H. (2021). ZEB1 Serves an Oncogenic Role in the Tumourigenesis of HCC by Promoting Cell Proliferation, Migration, and Inhibiting Apoptosis via Wnt/β-Catenin Signaling Pathway. Acta Pharmacol. Sin. 10.1038/s41401-020-00575-3 PMC8463676 33514855

[B23] Lin X. Li A.-m. Li Y.-H. Luo R.-C. Zou Y.-J. Liu Y.-Y. (2020). Silencing MYH9 Blocks HBx-Induced GSK3β Ubiquitination and Degradation to Inhibit Tumor Stemness in Hepatocellular Carcinoma. Sig Transduct Target. Ther. 5, 13. 10.1038/s41392-020-0111-4 PMC7018736 32296025

[B24] Lindeboom R. G. H. Vermeulen M. Lehner B. Supek F. (2019). The Impact of Nonsense-Mediated mRNA Decay on Genetic Disease, Gene Editing and Cancer Immunotherapy. Nat. Genet. 51, 1645–1651. 10.1038/s41588-019-0517-5 31659324 PMC6858879

[B25] Llovet J. M. Kelley R. K. Villanueva A. Singal A. G. Pikarsky E. Roayaie S. (2021). Hepatocellular Carcinoma. Nat. Rev. Dis. Primers 7, 6. 10.1038/s41572-020-00240-3 33479224 PMC13537433

[B26] Lombardi S. Testa M. F. Pinotti M. Branchini A. (2020). Molecular Insights into Determinants of Translational Readthrough and Implications for Nonsense Suppression Approaches. Ijms 21, 9449. 10.3390/ijms21249449 PMC7764779 33322589

[B27] Lu C. Rong D. Zhang B. Zheng W. Wang X. Chen Z. (2019). Current Perspectives on the Immunosuppressive Tumor Microenvironment in Hepatocellular Carcinoma: Challenges and Opportunities. Mol. Cancer 18, 130. 10.1186/s12943-019-1047-6 31464625 PMC6714090

[B28] McGlynn K. A. Petrick J. L. El‐Serag H. B. (2021). Epidemiology of Hepatocellular Carcinoma. Hepatology 73 (Suppl. 1), 4–13. 10.1002/hep.31288 PMC7577946 32319693

[B29] Muscella A. Vetrugno C. Cossa L. G. Marsigliante S. (2020). TGF‐β1 Activates RSC96 Schwann Cells Migration and Invasion through MMP‐2 and MMP‐9 Activities. J. Neurochem. 153, 525–538. 10.1111/jnc.14913 31729763

[B30] Nagy Á. Munkácsy G. Győrffy B. (2021). Pancancer Survival Analysis of Cancer Hallmark Genes. Sci. Rep. 11, 6047. 10.1038/s41598-021-84787-5 33723286 PMC7961001

[B31] Nasif S. Contu L. Mühlemann O. (2018). Beyond Quality Control: The Role of Nonsense-Mediated mRNA Decay (NMD) in Regulating Gene Expression. Semin. Cel Dev. Biol. 75, 78–87. 10.1016/j.semcdb.2017.08.053 28866327

[B32] Nogueira G. Fernandes R. García-Moreno J. F. Romão L. (2021). Nonsense-mediated RNA Decay and its Bipolar Function in Cancer. Mol. Cancer 20, 72. 10.1186/s12943-021-01364-0 33926465 PMC8082775

[B33] Pei Y. Zhang T. Renault V. Zhang X. (2009). An Overview of Hepatocellular Carcinoma Study by Omics-Based Methods. Acta Biochim. Biophys. Sinica 41, 1–15. 10.1093/abbs/gmn001 19129945

[B34] Popp M. W. Maquat L. E. (2018). Nonsense-mediated mRNA Decay and Cancer. Curr. Opin. Genet. Dev. 48, 44–50. 10.1016/j.gde.2017.10.007 29121514 PMC5869107

[B35] Roessler S. Jia H.-L. Budhu A. Forgues M. Ye Q.-H. Lee J.-S. (2010). A Unique Metastasis Gene Signature Enables Prediction of Tumor Relapse in Early-Stage Hepatocellular Carcinoma Patients. Cancer Res. 70, 10202–10212. 10.1158/0008-5472.CAN-10-2607 21159642 PMC3064515

[B36] Shaheen R. Anazi S. Ben-Omran T. Seidahmed M. Z. Caddle L. B. Palmer K. (2016). Mutations in SMG9, Encoding an Essential Component of Nonsense-Mediated Decay Machinery, Cause a Multiple Congenital Anomaly Syndrome in Humans and Mice. Am. J. Hum. Genet. 98, 643–652. 10.1016/j.ajhg.2016.02.010 27018474 PMC4833216

[B37] Subramanian A. Tamayo P. Mootha V. K. Mukherjee S. Ebert B. L. Gillette M. A. (2005). Gene Set Enrichment Analysis: a Knowledge-Based Approach for Interpreting Genome-wide Expression Profiles. Proc. Natl. Acad. Sci. 102, 15545–15550. 10.1073/pnas.0506580102 16199517 PMC1239896

[B38] Sukowati C. H. C. Anfuso B. Crocé L. S. Tiribelli C. (2015). The Role of Multipotent Cancer Associated Fibroblasts in Hepatocarcinogenesis. BMC Cancer 15, 188. 10.1186/s12885-015-1196-y 25879842 PMC4389787

[B39] Teng J. Wang Z.-Y. Prossnitz E. R. Bjorling D. E. (2008). The G Protein-Coupled Receptor GPR30 Inhibits Human Urothelial Cell Proliferation. Endocrinology 149, 4024–4034. 10.1210/en.2007-1669 18467434 PMC2488207

[B40] Tong H. Liu X. Li T. Qiu W. Peng C. Shen B. (2020). NR1D2 Accelerates Hepatocellular Carcinoma Progression by Driving the Epithelial-To-Mesenchymal Transition. Ott 13, 3931–3942. 10.2147/OTT.S237804 PMC7217318 32440156

[B41] Wang D. Zavadil J. Martin L. Parisi F. Friedman E. Levy D. (2011). Inhibition of Nonsense-Mediated RNA Decay by the Tumor Microenvironment Promotes Tumorigenesis. Mol. Cel Biol 31, 3670–3680. 10.1128/MCB.05704-11 PMC3165546 21730287

[B42] Wang H.-W. Hsieh T.-H. Huang S.-Y. Chau G.-Y. Tung C.-Y. Su C.-W. (2013). Forfeited Hepatogenesis Program and Increased Embryonic Stem Cell Traits in Young Hepatocellular Carcinoma (HCC) Comparing to Elderly HCC. BMC Genomics 14, 736. 10.1186/1471-2164-14-736 24160375 PMC3826595

[B43] Wang H. Chen X. Yang B. Xia Z. Chen Q. (2020). MiR-924 as a Tumor Suppressor Inhibits Non-small Cell Lung Cancer by Inhibiting RHBDD1/Wnt/β-Catenin Signaling Pathway. Cancer Cel Int 20, 491. 10.1186/s12935-020-01516-0 PMC7542747 33041671

[B44] Wurmbach E. Chen Y.-b. Khitrov G. Zhang W. Roayaie S. Schwartz M. (2007). Genome-wide Molecular Profiles of HCV-Induced Dysplasia and Hepatocellular Carcinoma. Hepatology 45, 938–947. 10.1002/hep.21622 17393520

[B45] Yamashita A. Izumi N. Kashima I. Ohnishi T. Saari B. Katsuhata Y. (2009). SMG-8 and SMG-9, Two Novel Subunits of the SMG-1 Complex, Regulate Remodeling of the mRNA Surveillance Complex during Nonsense-Mediated mRNA Decay. Genes Dev. 23, 1091–1105. 10.1101/gad.1767209 19417104 PMC2682953

[B46] Yin Z. Dong C. Jiang K. Xu Z. Li R. Guo K. (2019). Heterogeneity of Cancer-Associated Fibroblasts and Roles in the Progression, Prognosis, and Therapy of Hepatocellular Carcinoma. J. Hematol. Oncol. 12, 101. 10.1186/s13045-019-0782-x 31547836 PMC6757399

[B47] Yin Z. Jiang K. Li R. Dong C. Wang L. (2018). Multipotent Mesenchymal Stromal Cells Play Critical Roles in Hepatocellular Carcinoma Initiation, Progression and Therapy. Mol. Cancer 17, 178. 10.1186/s12943-018-0926-6 30593276 PMC6309092

[B48] Zheng K. He Z. Kitazato K. Wang Y. (2019). Selective Autophagy Regulates Cell Cycle in Cancer Therapy. Theranostics 9, 104–125. 10.7150/thno.30308 30662557 PMC6332805

[B49] Zhu L. Li L. Qi Y. Yu Z. Xu Y. (2019). Cryo-EM Structure of SMG1-SMG8-SMG9 Complex. Cell Res 29, 1027–1034. 10.1038/s41422-019-0255-3 31729466 PMC6951342

